# Prospective and Longitudinal Analysis of Lymphocyte Subpopulations in SARS-CoV-2 Positive and Negative Pneumonia: Potential Role of Decreased Naïve CD8^+^ in COVID-19 Patients

**DOI:** 10.3390/v17010041

**Published:** 2024-12-30

**Authors:** Makhabbat Bekbossynova, Lyudmila Akhmaltdinova, Kuanysh Dossybayeva, Ainur Tauekelova, Zauresh Smagulova, Tatyana Tsechoeva, Gulsimzhan Turebayeva, Aliya Sailybayeva, Zhanar Kalila, Takhmina Mirashirova, Timur Muratov, Dimitri Poddighe

**Affiliations:** 1 National Research Cardiac Surgery Center, Astana 010000, Kazakhstan; 2 School of Medicine, Nazarbayev University, Astana 010000, Kazakhstan; 3 Department of Infectious Diseases and Clinical Epidemiology, Astana Medical University, Astana 010000, Kazakhstan; 4 Department of Public Health of Astana, Astana 010000, Kazakhstan; 5 University Medical Center (UMC), Astana 010000, Kazakhstan; 6 College of Health Sciences, VinUniversity, Gia Lam District, Hanoi 100000, Vietnam

**Keywords:** pneumonia, SARS-CoV-2, COVID-19, lymphocyte immunophenotype, naïve CD8^+^ T cells

## Abstract

**Background**: During the acute phase of COVID-19, a number of immunological abnormalities have been reported, but few studies longitudinally analyzed the specific subsets of peripheral blood lymphocytes. **Methods**: In this observational, prospective, and longitudinal study, adult patients developing acute pneumonia during the COVID-19 pandemic have been followed up for 12 months. Peripheral blood lymphocyte subsets were assessed (with a specific focus on the memory markers) at 6 time points after the disease onset until 12 months. **Results**: A total of 76 patients with acute pneumonia (characterized by a prevalently interstitial pattern of lung inflammation) at the hospital admission (who completed the 12-month follow-up period) were recruited in this study. They were divided into two groups, namely positive (n = 31) and negative (n = 45) patients for the SARS-CoV-2 PCR test. In the acute phase, the general lymphocyte immunophenotyping profile was comparable for most parameters between these groups, except for B cells. When B and T cells were analyzed according to the expression of memory markers, a significant decrease in naïve CD8^+^ T cells was observed in the SARS-CoV-2-positive pneumonia group during the acute phase. Notably, this aspect was maintained during the follow-up period for at least 9 months. **Conclusions**: COVID-19 pneumonia seems to be associated with a lower number of naïve CD8^+^ T cells compared to pneumonia patients negative for this virus. This alteration can persist in the convalescent phase.

## 1. Introduction

The Coronavirus Disease 2019 (COVID-19) pandemic, due to the spread of the Severe Acute Respiratory Syndrome Coronavirus 2 (SARS-CoV-2) worldwide, has caused the most challenging health crisis of the current century so far. Since the beginning of the pandemic phase in 2020, almost 800 million COVID-19 cases and around 7 million COVID-19-related deaths have been recorded [1,2]. Although several vaccines against SARS-CoV-2 are currently available and despite the increased natural immunity deriving from previous infections, the emergence of new variants has perpetuated the viral circulation and created an endemic situation with periodical and/or seasonal outbreaks. Indeed, the rapid mutation rate of this virus enables it to evade the antibody and, more generally, the immune response of the host [3]. Therefore, even if the emergency phase of this global health threat is over, the medical problems related to COVID-19 are still present and represent a daily challenge for the whole medical community and health systems [4].

Although the therapeutic management of the acute phase of COVID-19 has been progressively improved and established, the long-term pathological, immunological, and medical consequences of this viral infection have not been fully elucidated and understood yet [5]. During the acute phase of SARS-CoV-2 infection, a number of immunological alterations have been reported, including several abnormalities in the proportion and/or homeostasis of different lymphocyte subsets in the peripheral blood [6]. Some authors noticed that T cell populations could be specifically altered, and some changes may correlate with the clinical outcomes of COVID-19 [7,8,9,10]. In our previous preliminary analysis, we also reported some lymphocyte (and, in detail, T-cell) alterations during the acute phase of COVID-19. Indeed, we observed a general perturbation inside the memory T cell compartment, which was more pronounced inside the CD8^+^ T cell subset. These changes were actually detected in both SARS-CoV-2-positive and negative patients affected with acute pneumonia characterized by a prevalently interstitial pattern of inflammation [11].

In this study, we longitudinally analyzed the peripheral lymphocytes from the acute phase until the end of a 12-month follow-up, in order to assess the mid/long-term impact of COVID-19 on the homeostasis of circulating lymphocyte subpopulations, compared to patients affected with SARS-CoV-2 negative pneumonia. This information could provide further insights into the immunological peculiarities of COVID-19, which may play a role in determining the clinical course of this infection.

## 2. Materials and Methods

### 2.1. Study Design and Patients

This observational, prospective, and longitudinal study included adult patients (aged ≥ 18 years) admitted to the hospital because of a diagnosis of acute pneumonia due to confirmed or suspected COVID-19 in the period between 14 February and 15 June 2022. All the study participants were admitted to the Department of Infectious Diseases (Sub-intensive and Intensive Care Units) of the City Infectious Diseases Center (CIDC) in Astana (Kazakhstan). Moreover, in order to be included in the present research, all patients must have attended the scheduled follow-up visits until 12 months after their hospital admission related to the acute respiratory disease (T0). In detail, all the study participants received a follow-up assessment at the following time points after the initial hospital admission date (T0): two weeks (T1), four weeks (T2), three months (T3), six months (T4), nine months (T5), and twelve months (T6).

The following exclusion criteria were established: lack of informed consent to participate in this study; anamnestic and/or clinical evidence of any pre-existing interstitial lung disease; presence of specific comorbidities and/or special conditions (including oncological disorders, hematological disorders, HIV infection, primary/secondary immunodeficiencies, chronic renal failure under dialysis, any disease previously treated with biological or immunosuppressive therapy, previous treatment with intravenous immunoglobulin and/or plasma derivatives, and pregnancy).

### 2.2. Ethical Statement

This study was conducted according to the guidelines of the Declaration of Helsinki and approved by the local Ethics Committee of the NRCCS (protocol no. 01-91/2021 dated 22 April 2021) in agreement with the ethical principles of the State Standard for Good Clinical Practice, Rules for Conducting Biomedical Research, and Requirements for Research Centers in Kazakhstan (released on 21 December 2020, № KR DSM-310/2020). Written and signed informed consent was obtained from all patients involved in the study (or legally authorized guardians, if appropriate).

### 2.3. Clinical, Laboratory, and Radiological Data

At the hospital admission, all the patients underwent a complete clinical examination, first-level blood work-up (including the main inflammatory parameters and biochemistry), and chest computerized tomography (chest CT performed by Ingenuity CT, Philips Healthcare, Cleveland, OH, USA). COVID-19 was diagnosed according to the World Health Organization COVID-19 case definitions and, accordingly, all these patients tested positive for SARS-CoV-2 RT-PCR (henceforth abbreviated as PCR). Patients with acute pneumonia who finally had a negative (SARS-CoV-2) PCR, were initially admitted to the hospital as suspected or probable COVID-19 cases. Therefore, at the hospital admission, all these patients with respiratory disease were classified in terms of COVID-19 severity according to the WHO criteria [12,13]. As regards the COVID-19 diagnostic work-up, all patients were tested by (SARS-CoV-2) PCR test at the hospital admission or before being transferred there; they were tested in different laboratories according to the referral pathway and national protocol in force during the study period (and, in general, during the pandemic), but all these tests were conducted by using certified diagnostic kits in authorized laboratories by the Ministry of Health of the Republic of Kazakhstan (according to the decree № KR DSM-114 from 12 November 2021). Moreover, all study participants were tested for specific SARS-CoV-2 serological titers (by chemiluminescent immunoassay, CLIA): in detail, anti-SARS-CoV-2 IgM (iFlash-SARS-CoV-2 IgM, Shenzhen YHLO Biotech Co. Ltd. (Shenzhen, China), cut-off 10 AU/mL), and anti-SARS-CoV-2 IgG (iFlash-SARS-CoV-2 IgG-S, Shenzhen YHLO Biotech Co., Ltd., cut-off 10 AU/mL, measuring range 2.00–3500 AU/mL) detect IgG specific for the both nucleocapsid and spike proteins. Additionally, anti-S-RBD SARS-CoV-2 Neutralization Antibody (NAb) (iFlash-2019-nCoV NAb, Shenzhen YHLO Biotech Co., Ltd., cut-off 10 AU/mL, measuring range 4.00–800 AU/mL), which is directed against the receptor-binding domain (RBD) of SARS-CoV-2 spike protein, was measured.

### 2.4. Flow Cytometry Analysis

All the study participants gave their consent for donating a small amount of blood for additional flow cytometry analyses due to the specific purposes of this research. The analysis of lymphocyte subpopulations by flow cytometry was performed on the same day when the blood samples were collected. Peripheral blood mononuclear cells (PBMCs) were isolated from EDTA whole blood (3–4 mL) by using Ficoll-Paque PLUS (Cytiva, Marlborough, MA, USA) density gradient centrifugation. The remaining erythrocytes were lysed with ACK lysing buffer (Gibco TM, ThermoFisher, Waltham, MA, USA). The following conjugated monoclonal antibodies were used in three different combinations (panels) to, respectively, investigate general lymphocyte, T cell, and B cell subpopulations: CD3-APC-A750 (UCHT1), CD4-APC (13B8.2), CD8-APC-A700 (B9.11), CD16-PB (3G8), CD19-PC5,5 (J3-119), CD21-PB (BL13), CD27-PC7 (1A4CD27), CD28-PC5.5 (CD28.2), CD38-APC-A750 (LS198-4-3), CD45-KrO (J33), CD45RO-FITC (UCHL1), CD56-PC5.5 (N901), CD95-PE (UB2), CD183-AF488 (G025H7), CD197-PC7 (G043H7), IgD-PE (IA6-2), IgM-APC (SA-DA4) (Immunotech SAS, Beckman Coulter, Marseille, France). The stained cells were resuspended in a staining medium and examined using DX Flex flow cytometer (Beckman Coulter, Brea, CA, USA). The resulting data were analyzed using CytoFlex, Kaluza 2.1 (Beckman Coulter, Brea, CA, USA). Forward and side scatter was used to clearly distinguish the lymphocyte population in addition to the signal from CD45 in all three panels that were used to analyze the general lymphocyte subpopulations (B cells, T cells, and NK cells), T cell subsets (with focus on naïve T, effector T, central memory T, and effector memory T cells), and B cell subsets (with focus on naïve B, unswitched memory B, switch memory B, and “double negative” B cells). The gating strategy is graphically summarized and shown in Supplementary Figure S1.

### 2.5. Statistical Analysis

Data collection and descriptive analysis were carried out by Microsoft^®^ Excel 2010 for Windows. Differences between the two groups for categorical variables were statistically analyzed using Fisher’s exact test. Differences between two groups for continuous variables were analyzed through the Kolmogorov–Smirnov test after excluding a normal distribution of values through the Shapiro–Wilk normality test. Accordingly, continuous variables were described as median and interquartile range (between 25th and 75th percentiles). The paired analysis between groups was performed through the Wilcoxon matched-pairs signed rank test. A *p*-value < 0.05 was considered statistically significant. The statistical analysis and graphics elaboration were performed using Prism 9 for MacOS (version 9.3.1, GraphPad Software).

## 3. Results

### 3.1. Demographic and Clinical Description

In this study, 76 patients (who were recruited during their hospital admission because of acute pneumonia consistent with COVID-19 and completed the 12-month follow-up period) were included. Their age ranged from 18.7 to 78.4 years; the majority of patients were male (n = 49), with a sex ratio equal to 1.8.

According to the (SARS-CoV-2) PCR, the study participants were divided into two groups, namely pneumonia patients with positive and negative tests (abbreviated, respectively, as Pn+PCR+ and Pn+PCR−). Their main demographic and clinical characteristics are summarized in Table 1.

While there was no difference in terms of sex distribution, these groups showed a statistically significant difference in terms of age: in detail, Pn+PCR+ patients were older than Pn+PCR− patients (61.6 vs. 47.8 years; *p* < 0.001). Nevertheless, these groups were comparable as regards the most relevant comorbidities associated with COVID-19 severity (such as cardiovascular disorders, diabetes mellitus type 2, and chronic renal disease). Notably, there was no significant difference in terms of previous SARS-CoV-2 vaccination between these groups, although the Pn+PCR− group showed a higher vaccination rate compared to Pn+PCR+ patients (55.5% vs. 38.7%, respectively). In terms of clinical severity (according to the WHO COVID-19 guidelines) [13], the large majority of patients were classified as moderate forms, except for ten patients who were in a severe/critical condition (Pn+PCR+: n = 7; Pn+PCR−: n = 3; *p* = ns), without any statistically significant difference between the two study groups. Similarly, there was no difference between these two groups in terms of relative parenchymal involvement based on lung CT estimation, which corresponded to 16% [4;28] and 20% [8.5;33] in the Pn+PCR+ and Pn+PCR− groups (*p* = ns), respectively.

**Table 1:** Main demographic and clinical parameters of the study groups.

	Pn+PCR+ (n = 31)	Pn+PCR− (n = 45)	*p*-Value
Sex (M:F)	22:9	27:18	0.4600
Age (yrs.)	61.6 [58.3–70.6]	47.8 [36.3–60.8]	0.0002
BMI	29.3 [25.0–34.6]	29.4 [22.9–33.6]	0.6600
SARS-CoV-2 Vaccination (y:n)	12:19	25:20	0.1600
**Main Comorbidity**			
CVD (y:n)	20:11	22:23	0.2414
Ischemic CVD (y:n)	3:28	2:43	0.3928
Hypertension (y:n)	20:11	20:25	0.1049
DMT2 (y:n)	5:26	8:37	0.9999
Renal Disease (y:n)	1:30	3:42	0.6412

Abbreviations: M, male; F, female; yrs., years; y, yes; n, no; CVD, cardiovascular disease; DMT2, diabetes mellitus type 2.

### 3.2. SARS-CoV-2 Serological Status

At the admission to the hospital, all study participants were tested for SARS-CoV-2 serological titers, including specific SARS-CoV-2 IgM, specific SARS-CoV-2 IgG, and specific IgG against the viral spike protein of SARS-CoV-2. As described in Table 2, in addition to being different in PCR results by definition, these two groups also showed statistically significant differences in their serological profiles, which were consistent with their respective group designations (namely, Pn+PCR+ or Pn+PCR−).

In fact, whereas Pn+PCR+ were characterized by positive and higher IgM titers (SARS-CoV-2 IgM; *p* < 0.001), Pn+PCR− patients showed much higher antibody levels against SARS-CoV-2 in terms of specific IgG (SARS-CoV-2 IgG; *p* < 0.05), including those IgG against the spike protein (SARS-CoV-2 IgG RBD; *p* < 0.001).

**Table 2:** Main laboratory parameters of the study groups in the acute phase of respiratory disease.

Parameter	Reference	Pn+PCR+ (n = 31)	Pn+PCR− (n = 45)	*p*-Value
SARS-CoV-2 IgM (AU/mL)	<10.0	1.5 [0.5–8.7]	0.4 [0.3–0.8]	**0.0002**
SARS-CoV-2 IgG (AU/mL)	<10.0	236.0 [80.9–1271.0]	881.3 [410.2–1407.0]	**0.0122**
SARS-CoV-2 IgG RBD (AU/mL)	<10.0	13.3 [7.1–378.3]	330.5 [68.1–772.3]	**0.0003**
WBC (×10^3^/µL)	5.0–10.0	6.4 [4.9–8.9]	8.8 [6.9–11.7]	**0.0009**
Neutrophils (×10^3^/µL)	2.5–8.0	4.0 [3.0–6.3]	5.6 [4.0–8.7]	**0.0104**
Lymphocytes (×10^3^/µL)	1.0–4.0	1.7 [1.3–2.2]	2.0 [1.3–2.6]	**0.0154**
Monocytes (×10^3^/µL)	0.1–0.7	0.6 [0.4–0.9]	0.9 [0.6–1.1]	0.0536
NLR	1.0–3.0	2.7 [2.0–4.2]	2.5 [1.7–5.9]	0.4412
Hemoglobin (g/dL)	13.5–18.0	12.7 [11.9–13.7]	13.1 [11.9–13.8]	0.8273
Thrombocytes (×10^3^/µL)	150–400	248 [195–312]	308 [231.5–385.5]	0.0920
CRP (mg/dL)	<1.0	0.9 [0.5–3.9]	0.8 [0.3–5.3]	0.7211
ESR (mm/h)	≤15	24.5 [18.5–35.5]	25.0 [13.5–32.5]	0.5419
Ferritin (ng/mL)	12–300	205.5 [93.3–348.2]	177.5 [65.0–322.9]	0.9320
Fibrinogen (g/L)	2.0–4.0	3.9 [3.6–5.4]	4.6 [3.4–5.8]	0.5169
D-Dimer (mg/L)	<0.4	0.3 [0.2–0.4]	0.3 [0.2–0.5]	0.5617

Abbreviations: WBC, white blood cells; NLR, neutrophil–lymphocyte ratio; CRP, c-reactive protein; ESR, erythrocyte sedimentation rate.

### 3.3. Main Hematological and Inflammatory Parameters in the Acute Phase of Respiratory Disease

Overall, both groups showed normal or mildly increased inflammatory parameters (which is consistent with viral pneumonia and its moderate severity on average) without showing any significant difference between them. Compared to the reference values, the count of white blood cells was not increased in either group, but Pn+PCR+ patients displayed a lower number of leukocytes (*p* < 0.001) than Pn+PCR− patients due to a reduction in both neutrophils (*p* < 0.05) and lymphocytes (*p* < 0.05) in the former group. All these data are summarized in Table 2. Probably, this observation further supports a different etiological background between these groups of patients, although they were both affected with pneumonia of similar clinical severity and lung involvement, as mentioned above.

### 3.4. Analysis of Lymphocyte Subsets in the Acute Phase of Respiratory Disease

The general lymphocyte immunophenotyping is displayed in Table 3. Pn+PCR+ patients showed a significantly lower number of B cells compared to Pn+PCR− patients, which is evident in both percentages (0.151 [0.097–0.278]% vs. 0.280 [0.196–0.454]% of total lymphocytes; *p* < 0.01, respectively) and absolute counts (9.61 [6.79–13.15] × 10^3^/µL vs. 14.05 [10.65–19.98] × 10^3^/µL; *p* < 0.01, respectively). All other general lymphocyte subsets were not significantly different between these two groups, except for CD8^+^ cells, which showed a mild statistical difference between Pn+PCR+ and Pn+PCR−, but only in their absolute count (0.319 [0.243–0.479] × 10^3^/µL vs. 0.469 [0.280–0.656] × 10^3^/µL; *p* < 0.05, respectively).

Table 4 summarizes the memory phenotypes of T cells. Notably, these two groups of pneumonia patients showed no significant differences, except for the number of naïve CD8^+^ cells, which were markedly lower in Pn+PCR+ patients than in the Pn+PCR− group in both absolute (0.047 [0.026–0.110] × 10^3^/µL vs. 0.155 [0.063–0.277] × 10^3^/µL; *p* < 0.01, respectively) and percentage (relative) terms (19.39 [9.55–29.98]% vs. 31.97 [19.25–49.63]% of total lymphocytes; *p* < 0.05).

As regards B cells (whose memory immunophenotyping is shown in Table 4), no statistically significant differences between groups were observed, except for naïve B cells, which showed a lower absolute (but not percentage) count in Pn+PCR+ patients compared to the other group (0.075 [0.053–0.157] × 10^3^/µL vs. 0.145 [0.093–0.278] × 10^3^/µL; *p* < 0.05, respectively).

**Table 4:** Lymphocyte immunophenotyping in the acute phase of respiratory disease, according to the expression of memory markers (percentage values to the respective parental population, indicated in bold characters).

Cell Markers	T Cell Subsets	Pn+PCR+ (n = 31)	Pn+PCR− (n = 45)	*p*-Value
CD4^+^ T Cells				
CCR7 + CD45RO− (×10^3^/µL)	*Naive*	0.246 [0.121–0.432]	0.320 [0.218–0.494]	0.1038
CCR7 + CD45RO− (%)		35.16 [26.20–48.03]	39.06 [31.68–48.16]	0.1632
CCR7 + CD45RO+ (×10^3^/µL)	*Central* *Memory*	0.230 [0.163–0.384]	0.256 [0.150–0.340]	0.8381
CCR7 + CD45RO+ (%)		33.21 [21.50–42.75]	25.51 [20.54–36.27]	0.1206
CCR7-CD45RO+ (×10^3^/µL)	*Effector* *Memory*	0.191 [0.119–0.235]	0.237 [0.108–0.322]	0.1298
CCR7-CD45RO+ (%)		24.52 [18.56–32.63]	25.74 [16.12–34.32]	0.8469
CCR7-CD45RO− (×10^3^/µL)	*Effector*	0.016 [0.008–0.035]	0.018 [0.009–0.033]	0.9968
CCR7-CD45RO− (%)		2.44 [1.12–3.89]	1.75 [1.36–2.99]	0.3193
**CD8^+^ T Cells**				
CCR7 + CD45RO− (×10^3^/µL)	*Naive*	0.047 [0.026–0.110]	0.155 [0.063–0.277]	**0.0036**
CCR7 + CD45RO− (%)		19.39 [9.55–29.98]	31.97 [19.25–49.63]	**0.0486**
CCR7 + CD45RO+ (×10^3^/µL)	*Central* *Memory*	0.023 [0.013–0.057]	0.039 [0.023–0.069]	0.1521
CCR7 + CD45RO+ (%)		10.16 [4.25–14.68]	9.23 [5.66–13.90]	0.4140
CCR7-CD45RO+ (×10^3^/µL)	*Effector* *Memory*	0.109 [0.067–0.133]	0.110 [0.061–0.180]	0.1070
CCR7-CD45RO+ (%)		34.71 [23.11–45.37]	28.46 [18.76–36.46]	**0.0314**
CCR7-CD45RO− (×10^3^/µL)	*Effector*	0.072 [0.058–0.169]	0.091 [0.052–0.192]	0.5486
CCR7-CD45RO− (%)		28.19 [20.48–39.09]	23.17 [14.45–35.34]	0.1678
**B Cells**				
IgD + CD27− (×10^3^/µL)	*Naive*	0.075 [0.053–0.157]	0.145 [0.093–0.278]	**0.0476**
IgD + CD27− (%)		62.24 [52.27–74.01]	69.19 [59.14–77.00]	0.2809
IgD + CD27+ (×10^3^/µL)	*Memory* *Unswitched*	0.012 [0.008–0.028]	0.024 [0.010–0.039]	0.0613
IgD + CD27+ (%)		9.59 [5.23–13.36]	8.90 [6.49–11.89]	0.6200
IgD-CD27+ (×10^3^/µL)	*Memory* *Switched*	0.028 [0.021–0.049]	0.039 [0.026–0.065]	0.3987
IgD-CD27+ (%)		21.84 [12.85–31.65]	17.28 [11.43–23.65]	0.2323
IgD-CD27− (×10^3^/µL)	*IgD-CD27* *double negative*	0.005 [0.004–0.007]	0.006 [0.004–0.013]	0.1904
IgD-CD27− (%)		3.64 [2.64–4.90]	3.17 [1.95–4.83]	0.3364

### 3.5. Longitudinal Analysis of Lymphocyte Subpopulations

**Figure 1:**
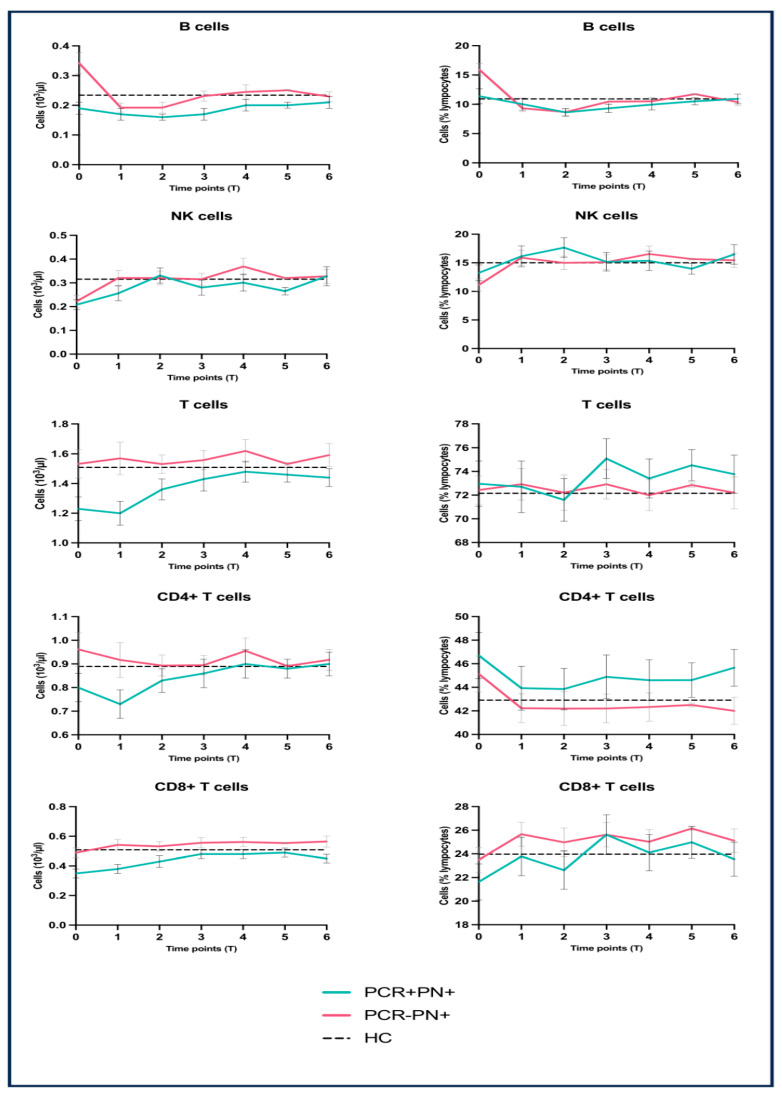
Longitudinal trend of the main lymphocyte subpopulations: percentage values refer to the total lymphocyte number (the points and related bars correspond to the mean and its standard errors, respectively; the dashed line represents the mean value observed in Kazakh healthy controls, as explained in the text).

The longitudinal analysis of the general lymphocyte populations is displayed in Figure 1: here, the trends of total B cells, NK cells, and the main T cell subsets (CD4^+^ and CD8^+^) are compared at all time points until 12 months after the acute phase of infection (characterized by pneumonia as per inclusion criteria). Moreover, each panel also shows the corresponding mean value observed in Kazakh healthy controls (as collected in our previous preliminary study) [11]. Indeed, in order to increase the graphical impact and perform an initial analysis of the longitudinal trends, the points and related bars correspond to the mean and its standard errors, respectively.

The significant difference in B cell numbers described in the acute phase (see previous subsection), based on the healthy control values, appears to be mainly due to their increase in the Pn+PCR− group during the acute phase. Indeed, in these patients, the number of B cells rapidly decreases after T0, so their difference with the Pn+PCR+ group disappears after two weeks (T1). The same analytical approach shows that NK cells tend to decrease in the acute phase for both groups compared to the controls line. Thus, no statistical difference between them was observed. Again, the NK cells return to normal values within two weeks (T1) and remain stable for the entire period of follow-up.

Although there is no statistically significant difference during the acute phase, Pn+PCR+ patients appeared to have a decreased absolute number of T cells compared to Pn+PCR−; this trend was also present in the absolute counts of both CD4^+^ and CD8^+^ T cells. However, this finding is not equally evident when these subpopulations are expressed as a percentage of total lymphocytes.

**Figure 2:**
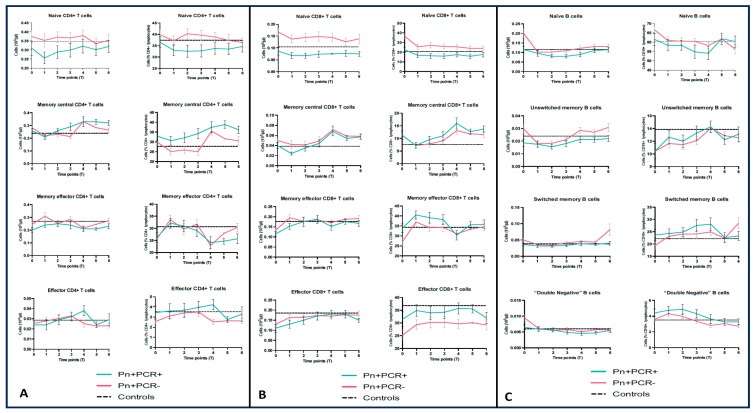
Longitudinal trend of the main lymphocyte sub-subpopulations, according to the expression of memory markers percentage values to the respective parental population, namely CD4^+^ T cells (**A**), CD8^+^ T cells (**B**), and B cells (**C**) (the points and related bars correspond to the mean and its standard errors, respectively; the dashed line represents the mean value observed in Kazakh healthy controls, as described in the text).

We drafted similar longitudinal graphics for each one of the three main lymphocyte subpopulations, namely CD4^+^ cells, CD8^+^ cells, and B cells (Figure 2A–C), in order to specifically analyze the naïve/memory profile of these lymphocytes. In this regard, as described in the previous subsection (related to the acute phase), the most significant and concordant (considering both absolute and relative counts) difference between Pn+PCR+ and Pn+PCR− patients was observed in the number of naïve CD8^+^ T cells, which were lower in the former group.

**Figure 3:**
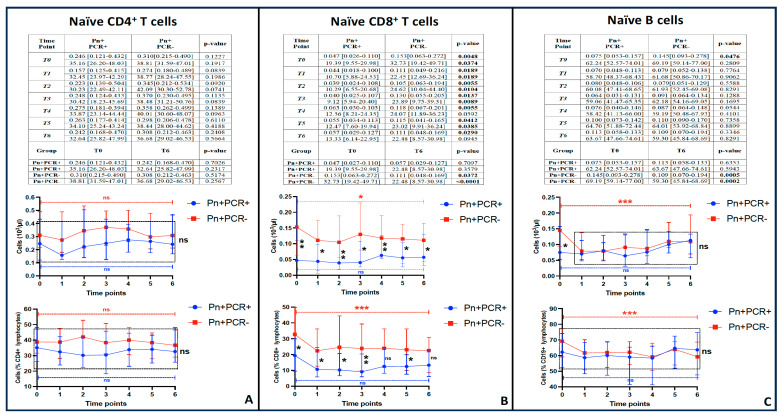
Longitudinal trend of the naïve T and B lymphocytes (for each time point, two values are displayed: absolute and percentage counts; percentage values to the respective parental population, respectively, CD4^+^ T cells (**A**), CD8^+^ T cells (**B**), and B cells (**C**)). All the curve values are reported as mean and interquartile range. The red and blue dashed lines between T0 and T6 indicate the statistical significance (or less) of their difference inside the same group (the color is consistent with the curve that each one refers to); the black dashed rectangle, wherever it is present, indicates the absence of statistical significance (ns) between the groups at each time point. The asterisks indicated the level of statistical significance (*: *p* < 0.05; **: *p* < 0.01; ***: *p* < 0.001).

Moreover, as shown in the corresponding Figure 3B, such a gap between these groups of patients was maintained (with statistical significance) for at least 9 months of the follow-up period. We also noticed that such a longitudinal trend of decreased naïve lymphocytes was present in CD4^+^ T cells and even in B cells, but it was less accentuated without reaching any statistical significance, as explained below.

### 3.6. Specific Analysis of Naïve Cells in the Three Main Lymphocyte Subpopulations

Overall, the graphical analysis of the longitudinal trends showed a gap between Pn+PCR+ and Pn+PCR− patients in terms of naïve CD8^+^ T cells, CD4^+^ T cells, and B cells, as displayed in Figure 3. As mentioned, this difference is more accentuated for naïve CD8^+^ T cells: indeed, such a difference is basically maintained at all time points with statistical significance until 9 months of follow-up. Moreover, the inside-group paired comparison between T0 (acute phase) and T6 (12-month follow-up) revealed a statistically significant difference in the number of naïve CD8^+^ T cells for Pn+PCR− patients, but not for Pn+PCR+ patients, suggesting that the former group may have had an increase in the number of these cells during the acute phase, compared to their personal basal levels (as measured at 12 months’ distance from the hospital admission), which did not occur in the Pn+PCR+ patients.

Notably, a similar situation also emerges from the same paired analysis performed for naïve B cells, even though the inter-group differences at each time point (with the Pn+PCR+ group showing lower levels of these cells than in the Pn+PCR− group) did not reach statistical significance.

Finally, a gap between these groups may be present for naïve CD4^+^ T cells, too, although it was more evident in the post-acute phases. However, this difference did not reach a persisting level of statistical significance over the follow-up period. Moreover, the inside-group paired analysis between T0 and T6 did not show any significant difference in Pn+PCR− patients for these cells, unlike naïve CD8^+^ T cells and naïve B lymphocytes.

## 4. Discussion

In this study, we analyzed the longitudinal trend of the main lymphocyte subsets in patients affected with acute pneumonia characterized by a prevalently interstitial pattern of inflammation. By comparing Pn+PCR+ and Pn+PCR− groups (patients affected with SARS-CoV-2-related and -unrelated pneumonia, respectively), we observed that their general lymphocyte immunophenotyping profile (B, T, and NK cells) was comparable in the acute phase and over the 12-month longitudinal course. In the acute phase, the only statistically significant difference was noticed in the absolute and relative counts of B cells, which were both lower in COVID-19 patients. However, based on the published reference values [14,15] and our previous reference values from local controls [11], this difference could be due to an increase in B cells during the acute phase in the Pn+PCR− group rather than their decrease in COVID-19 patients. With regards to T cells, a trend toward lower levels in COVID-19 pneumonia (compared to Pn+PCR− patients) was noticed, but without any statistical significance. The NK cell dynamics were similar between these two groups, even in the acute phase.

Therefore, considering that these groups were comparable for clinical (disease severity) and radiological (type and extension of lung damage) factors, these observations could suggest that most of the changes observed in the general lymphocyte profile might be related to the pathological setting of acute pneumonia (and not specifically linked to the specific infectious etiology and, in detail, to SARS-CoV-2 infection).

Several studies reported a general lymphocyte depletion, mainly expressed in the T cell compartment, during acute COVID-19, especially in severe cases [16,17,18,19]. However, in most studies, the term of comparison was represented by healthy controls only. Indeed, the study by Quian et al., including non-COVID-19 patients with acute respiratory disease, showed that the total lymphocyte number and T cell subset counts were similarly reduced in both influenza A and COVID-19 [20]. Moreover, a number of pre-pandemic studies also reported alterations of lymphocyte subpopulations, especially in the T cell subset, during influenza A, SARS-CoV-1, and other viral respiratory infections complicated with pneumonia [21,22,23,24].

COVID-19 patients have lower levels of B cells than those affected with (SARS-CoV-2) PCR negative pneumonia at the disease onset, which is a novel observation. Indeed, the aforementioned study by Qian et al. did not include any data about B cell homeostasis in the peripheral blood [21]. However, several studies showed that B cell numbers were in the normal range (as defined by respective healthy controls) in both severe and non-severe COVID-19 [25,26,27,28]. Moreover, Guo et al. clearly highlighted an increase in B cells in the acute phase of influenza H1N1, especially in severe cases [23]. Therefore, SARS-CoV-2 could differ from other respiratory viruses in eliciting or, more correctly, not eliciting a general and reactive quantitative B cell response in the circulating pool.

When we analyzed both B and T cells for memory markers, the most significant difference between these groups during the acute phase of the infection was represented by a reduction in naïve CD8^+^ T cells. However, looking at the longitudinal trend, such a decrease in naïve cells also seems to involve the other two main lymphocyte subpopulations (namely, B and CD4^+^ T cells); even though this gap does not reach the statistical significance for these cells, perhaps due to the small sample size. Whereas the significant difference in the absolute count of naïve B cells is limited to the disease onset, the lower levels of naïve CD8^+^ T cells are evident in both absolute and percentage counts and, notably, are maintained beyond the acute phase. Moreover, when we performed a paired analysis between the time points of the disease onset (T0) and the last follow-up visit after 12 months (T6), non-COVID-19 (PCR-Pn+) patients displayed a significant difference in the naïve CD8^+^ T cells between them, suggesting a “reactive” increase in these cells in the acute phase and, then, a return to their steady state over time. Conversely, during COVID-19 pneumonia (PCR+Pn+ group), naïve CD8^+^ T cells do not increase and actually maintain those reduced levels during the convalescence until at least 9 months.

A decrease in naïve CD8^+^ T cells has been noticed by other authors in the setting of COVID-19. Kwiecien et al. observed a lower proportion of naïve CD8^+^ T cells in COVID-19 patients with radiologically evident pneumonia but not in those without lung alterations at the chest X-ray [29]. Mann et al. also noticed a decline in peripheral blood naïve CD8^+^ T cells at the clinical onset of COVID-19 compared to controls [30]. Kreutmair et al. extensively analyzed the immunological characteristics of COVID-19 patients and compared them with patients developing hospital-associated pneumonia, in addition to some healthy controls. In terms of naïve T cells, their data showed lower values in naïve T CD8^+^ cells in COVID-19 patients compared to both other groups [31]. However, these studies were limited to the acute phase of COVID-19. Conversely, like in our research, Rajamanickam et al. described the longitudinal course of lymphocyte subsets after the acute phase and until at least six months after SARS-CoV-2 primary infection: they reported a trend toward lower counts of naïve T cells (both CD4^+^ and CD8^+^) persisting during the convalescence [32]. Notably, their study presented remarkable differences from ours, such as the fact that the study groups at different time points did not include the same patients’ cohort, and their analysis was focused on the comparison between mild and severe forms of COVID-19 [32]. Even though no significant differences in naïve B and T cells were actually observed between controls and COVID-19 patients in the study by Shuwa et al., these authors also described a progressive and decreasing trend of naïve CD8^+^ T cells from controls to severe COVID-19 cases, passing through mild and moderate forms; in this regard, it is interesting to notice that such a decrease in naïve CD8^+^ T cells was also present in convalescent patients [33]. Wiech et al. reported a time-dependent (3-month vs. 6-month follow-up, after COVID-19 onset) decrease in naïve CD8^+^ T cell percentage in all their groups of convalescent patients according to the severity of acute disease (mild, moderate, severe), although this relative depletion was more evident in patients recovering from severe forms [34].

Lymphocyte activation and recruitment of naïve lymphocytes could be one explanation for their decrease in the circulating pool. Indeed, in the post-mortem analysis of lungs from patients who died of severe COVID-19, it was reported a lung injury characterized by marked interstitial infiltration of lymphocytes [35,36]. A study by Puzyrenko et al. also highlighted the presence of a large component of CD8^+^ T cells in COVID-19 victims [37], but other comparative histopathological studies between COVID-19 and influenza showed that several features (including lymphocyte infiltrates in lungs) were similar, also in terms of CD8^+^ T cell tissue recruitment and CD8/CD4 ratio [38,39]. Therefore, increased peripheral tissue recruitment cannot entirely explain the differences in circulating naïve CD8^+^ T lymphocytes between COVID-19-related and -unrelated pneumonia. Moreover, this potential mechanism cannot be implicated in the persistent lower levels of naïve T CD8^+^ cells beyond the acute phase of the infection and pulmonary disease. It could be hypothesized that SARS-CoV-2 might have a greater impact on lymphocyte homeostasis than other respiratory viruses. Indeed, several studies (that also reported some persistent alterations in the lymphocyte profiles of convalescent COVID-19 patients) suggested that the circulating lymphocyte pool of SARS-CoV-2-infected patients displays more senescent status and/or exhaustion markers, which could also affect the availability and/or regeneration of naïve T cells, especially in patients recovering from moderate-severe forms of SARS-CoV-2 infections and/or developing long COVID-19 [34,40,41]. However, it is also plausible that such an “exhaustion” of naïve lymphocytes (and, in detail, T cells) could be also affected by patients’ age, considering that the older age of our COVID-19 patients was the only (clinical and demographic) significant difference, compared to Pn+PCR− study participants. Indeed, an age-related decline in T cell number has been described [42], and some authors also discussed the potential role of a more abundant T cell naïve pool in younger individuals as a factor mitigating SARS-CoV-2 infections [43,44,45].

Of course, there are several limitations that affected the present study. The number of study participants with a complete follow-up (that we could recruit for this research) was relatively small. Although the PCR test differentiated among SARS-CoV-2-positive and negative patients affected with interstitial pneumonia, we could not identify at that time any specific SARS-CoV-2 variants in the former group or the other viruses implicated in the respiratory disease in the latter group (no specific microbiological tests were performed to investigate other respiratory etiological agents).

In perspective, the identification of some general and persistent immunological alterations in COVID-19, such as a decrease in naïve CD8^+^ T cells in the present study and/or others from additional studies, could be used to better assess these patients during the acute phase and/or monitor their longitudinal course (with respect to the potential occurrence of long COVID-19, too). In this regard, if further, larger, and more standardized studies are carried out to evaluate those immunological alterations and their association-correlation with specific clinical aspects, potential biomarkers might be identified to provide more personalized medical management starting from the initial phases of COVID-19.

## 5. Conclusions

Acute and infectious pneumonia (with a prevalently interstitial pattern of lung inflammation) can be characterized by several alterations of the lymphocyte subpopulations. Overall, the impact of SARS-CoV-2 infection on peripheral blood lymphocytes is comparable to that seen in non-COVID-19 pneumonia, except for a few but potentially relevant aspects. In the present study, we observed lower levels of naïve cells at the disease onset, especially among CD8^+^ T cells. Notably, such a decrease in naïve CD8^+^ T cells in COVID-19 pneumonia patients (compared to those negative for SARS-CoV-2) was not limited to the acute phase of the infection but persisted in the convalescent phase. Further studies are needed to explore the immunopathological significance of this finding in both acute and long COVID-19.

## Figures and Tables

**Table 3:** General lymphocyte subpopulations of the study groups (percentage values refer to the total lymphocyte population) in the acute phase of respiratory disease.

Lymphocyte Subpopulation	Pn+PCR+ (n = 31)	Pn+PCR− (n = 45)	*p*-Value
B cells (×10^3^/µL)	0.151 [0.097–0.278]	0.280 [0.196–0.454]	**0.0017**
B cells (%)	9.61 [6.79–13.15]	14.05 [10.65–19.98]	**0.0048**
NK cells (×10^3^/µL)	0.174 [0.122–0.282]	0.161 [0.086–0.340]	0.3680
NK cells (%)	11.33 [7.41–18.30]	10.27 [5.00–16.26]	0.4642
T cells (10^3^/µL)	1.215 [0.835–1.621]	1.533 [0.986–1.863]	0.1186
T cells (%)	75.49 [64.15–81.01]	72.96 [65.77–79.55]	0.5566
CD4^+^ T cells (×10^3^/µL)	0.709 [0.489–1.099]	0.893 [0.566–1.207]	0.5022
CD4^+^ T cells (%)	45.33 [38.85–54.48]	45.46 [37.61–51.98]	0.9636
CD8^+^ T cells (×10^3^/µL)	0.319 [0.243–0.479]	0.469 [0.280–0.656]	**0.0351**
CD8^+^ T cells (%)	20.44 [13.89–25.25]	22.80 [17.14–27.98]	0.4322
DNT cells (×10^3^/µL)	0.030 [0.022–0.048]	0.045 [0.022–0.060]	0.5022
DNT cells (%)	2.20 [1.31–3.45]	2.10 [1.41–2.61]	0.2787
DPT cells (×10^3^/µL)	0.012 [0.007–0.022]	0.014 [0.007–0.022]	0.9944
DPT cells (%)	0.82 [0.43–1.31]	0.63 [0.34–1.25]	0.9168

## Data Availability

Data cannot be publicly released without patients’ consent for open, unrestricted access. Researchers who meet the criteria for research access to the data may contact the corresponding author for further information.

## Supplementary Materials

The following supporting information can be downloaded at: https://www.mdpi.com/article/10.3390/v17010041/s1, Figure S1: Graphical summary of the gating strategy.
