# Supplementary material for: Prospective and Longitudinal Analysis of Lymphocyte Subpopulations in SARS-CoV-2 Positive and Negative Pneumonia: Potential Role of Decreased Naïve CD8+ in COVID-19 Patients
Source: Viruses. 2024 Dec 30;17(1):41. doi: 10.3390/v17010041 (PMC11768816; doi:10.3390/v17010041)

Supplementary Figure S1. Graphical summary of the gating strategy (N: naïve; E: effector; CM: central memory; EM: effector memory; uM: unswitched memory; sM: switched memory; DN: double negative).

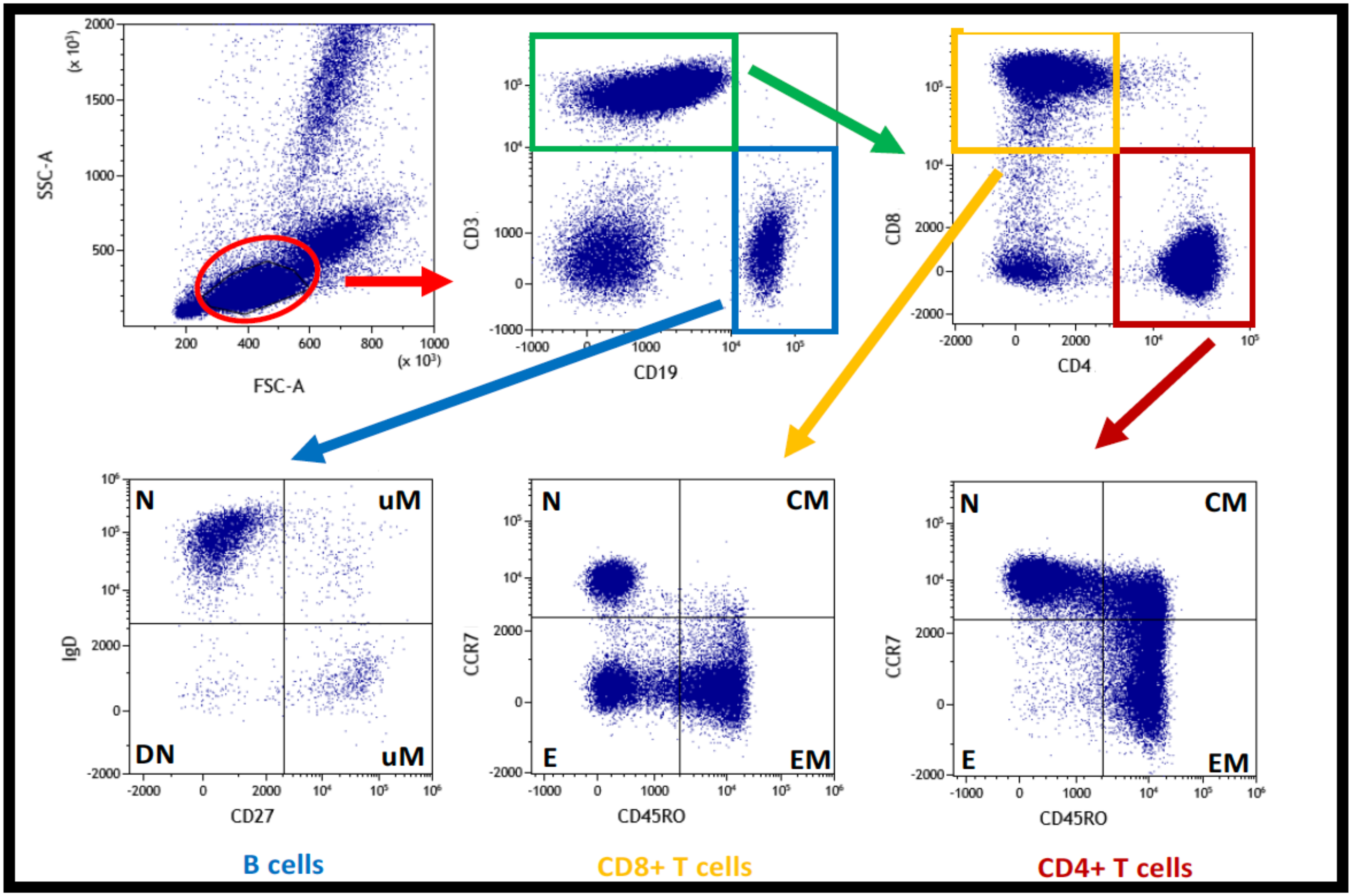

Supplement: Supplementary file 1 [file viruses-17-00041-s001.zip › viruses-3342912-supplementary.pdf]
